# Contemporaneous Insufficiency Fractures of Bilateral Femoral Necks in an Older Patient Taking Bisphosphonate: A Case Report and Literature Review

**DOI:** 10.1155/2022/9294289

**Published:** 2022-04-29

**Authors:** Hiroaki Tagomori, Nobuhiro Kaku, Shota Sato, Tsuguaki Hosoyama, Hiroshi Tsumura

**Affiliations:** Department of Orthopedic Surgery, Faculty of Medicine, Oita University, 1-1 Idaigaoka Hasama-machi, Yufu City, Oita 879-5593, Japan

## Abstract

We report, to the best of our knowledge, the first case of simultaneous insufficiency fracture of the bilateral femoral neck in an older patient taking bisphosphonate. Femoral neck fractures frequently occur in older individuals because of traumatic incidents, such as a fall. A 74-year-old woman with osteoporosis was taking raloxifene hydrochloride and bisphosphonate for approximately 5 and 3 years, respectively. Despite no history of falls or any other traumatic incidence, the patient reported cooccurrence of pain on both sides of the hip. Imaging revealed bilateral femoral neck fractures. Bipolar hemiarthroplasty and internal fixation were conducted on the right and left hips, respectively. At 6 months postoperatively, the patient reported gradual disappearance of left hip pain, and a radiograph revealed that the fracture had healed. Overall, clinical and histological findings suggested an atypical fracture, thereby proving that this type of fracture can occur in areas other than the femoral shaft.

## 1. Introduction

Fractures in older individuals are often caused by osteoporosis, and osteoporotic treatment has become a standard practice to prevent fractures. However, therapeutic agents including bisphosphonates (BP) are associated with complications, including osteonecrosis of the jaw and atypical femoral fractures [1].

Femoral neck fractures in older individuals are also attributed to osteoporosis and are frequently encountered in daily practice. These fractures affect the activities of daily living and increase mortality in older individuals [2] and are mostly caused by falls. Spontaneous stress fractures rarely occur. Severe osteoporosis can cause insufficiency fracture within the range of activities of daily living; however, such cases are sporadic. Additionally, the simultaneous occurrence of bilateral femoral neck fracture is an even rarer entity. Furthermore, bilateral fractures can rarely occur during the same period. Herein, we report a case of spontaneous bilateral femoral neck fractures in a patient taking BP for 3 years.

## 2. Case Presentation

Our patient was a 74-year-old woman with no history of surgery or fractures in the lower extremities. She had no other medical history and was not undergoing treatment for any diseases. However, she was taking 60 mg/day raloxifene hydrochloride and 75 mg/month sodium risedronate hydrate for osteoporosis for the past 5 and 3 years, respectively. Although she had not been in the habit of taking walks, she had started walking for 30 min daily for 5 months for her health before the appearance of pain. She was diagnosed with bilateral stress fractures of the femoral neck and was instructed to rest without other treatment. However, as her symptoms did not improve, she came to our hospital 2 months after the onset of symptoms.

The patient's height and weight were 151 cm and 43 kg, respectively (body mass index, 18.9 kg/m^2^). She had pain-avoiding claudication with no range of motion limitation, but she had tenderness in both Scarpa triangles. The Harris hip score was 45/55 (right/left). Bone mineral densities of the femoral neck and lumbar spine were 0.597/0.463 (Rt/Lt) and 0.670 g/cm^2^, respectively. Blood test findings were as follows: calcium, 9.34 mg/dL (normal range, 8.8–10.1); phosphorus, 3.74 mg/dL (2.7–4.6); alkaline phosphatase, 138 U/L (106–322); tartrate-resistant acid phosphatase-5b, 289 mU/dL (170–590); intact type I procollagen N-terminal propeptide, 15.6 *μ*g/L (18.1–74.1); and 25-OH vitamin D, 15.4 ng/mL (10–30).

**Figure 1:**
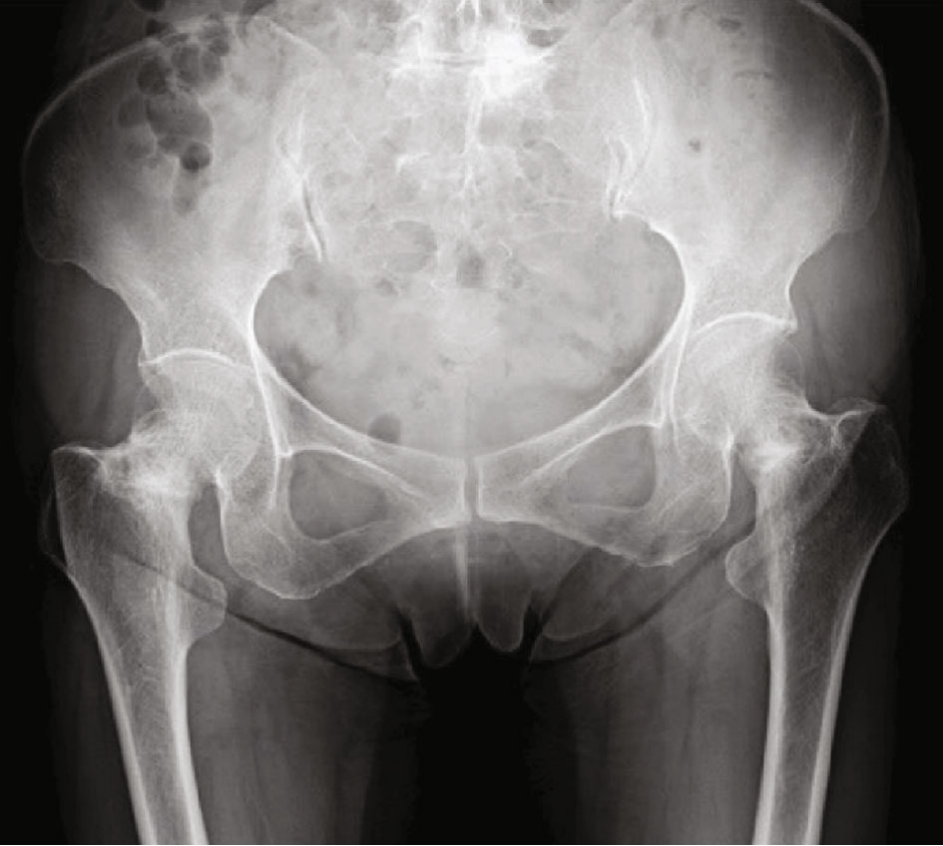
Preoperative radiograph of the present case demonstrating bilateral femoral neck stress fracture with osteosclerosis.

**Figure 2:**
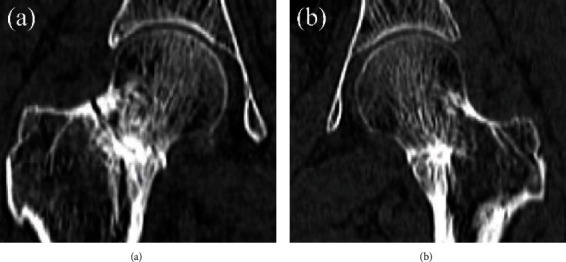
Computed tomography coronal image demonstrating a circumferential fracture and surrounding osteosclerosis of the femoral neck on the right side (a) and the medial fracture on the left side (b).

Radiographs revealed osteosclerosis on the medial side of the bilateral femoral neck. Her right side was deformed inwardly (Figure 1). Computed tomography revealed fracture lines and surrounding osteosclerosis around the entire femoral neck on the right side and medial fracture lines on the left side (Figures 2(a) and 2(b)). The neck angles of the femur were 125° on the right side and 129° on the left side, whereas the anterior femoral torsion angles were 20° on the right side and 30° on the left side.

**Figure 3:**
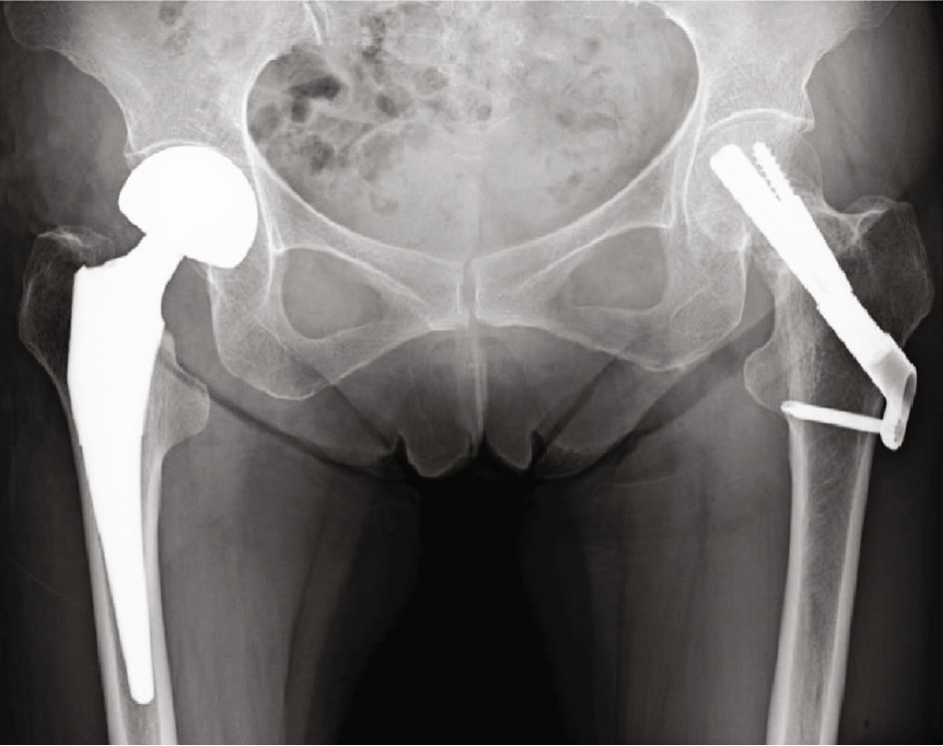
Postoperative radiograph demonstrating the treatment with bipolar hemiarthroplasty of the femoral neck on the right side and with osteosynthesis on the left side.

**Figure 4:**
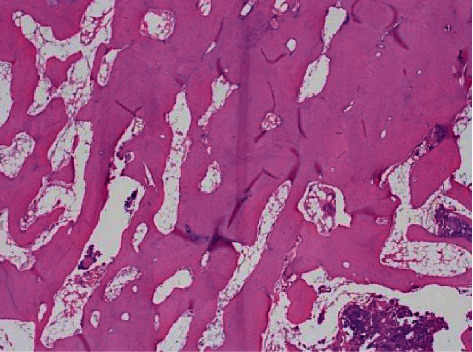
Histology of the fracture site. An increase in the empty lacunae is observed in the thickened bony trabeculae under low magnification of the bone sample around the fracture site (100x).

**Figure 5:**
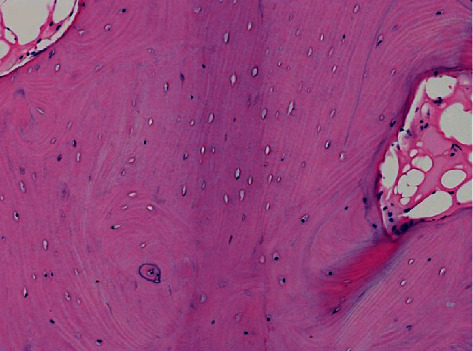
Histology of the fracture site. In the high magnification of the region (200x), thickening of the bony trabeculae with few cells on the surface is observed in the fracture site.

As the displacement occurred between bone fragments on the right side of the femur, bipolar hemiarthroplasty was performed to hold the loaded limb during the transfer postoperatively (Figure 3). The left side demonstrated no displacement between both bone fragments, and osteosynthesis was performed to preserve the native joint. The fractured part of the retrieved femoral head was discolored white and hard. During the surgery on the left side, significant osteosclerosis at the fracture site made it difficult to drill the lag screws. Histopathological findings demonstrated thickened bony trabeculae at the fracture site. Additionally, there were a few cells, such as osteoclasts, on the trabecular surface and many empty lacunae in the bony trabeculae (Figures 4 and 5).

**Figure 6:**
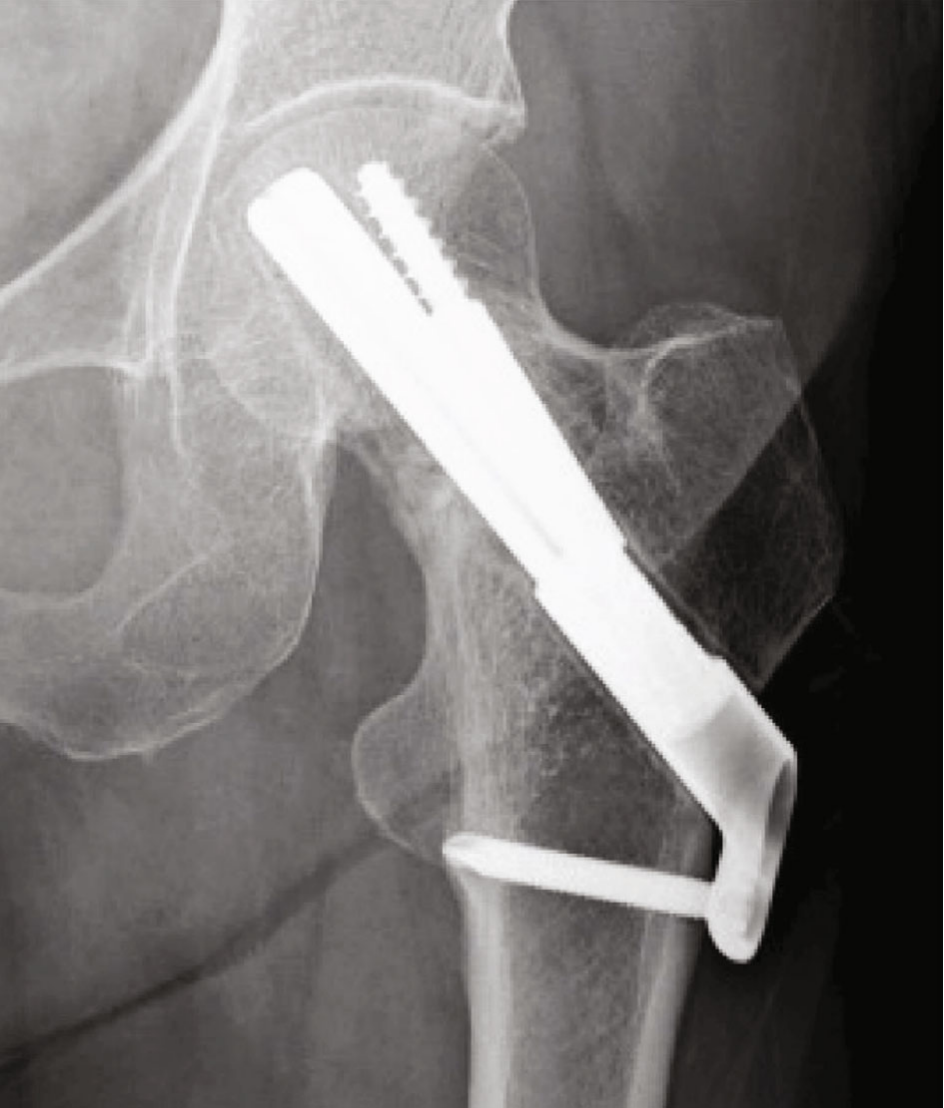
A left hip radiograph obtained at 1 year postoperatively demonstrating a radiolucent line around the implant that was not present immediately postoperatively.

Sodium risedronate hydrate was discontinued, and a subcutaneous injection with teriparatide (20 *μ*g/day) and eldecalcitol of active vitamin D (0.75 *μ*g/day) was started postoperatively. At 6 months postoperatively, the left hip pain gradually disappeared, and a radiograph revealed that the fracture had healed. The modified Harris hip score at 1 year postoperatively was 94/96 (right/left), and osteosclerosis of the fracture became less noticeable but persisted (Figure 6).

This case study was performed per the ethical standards of the 1964 Declaration of Helsinki and its later amendments. The approval of the Institutional Review Board of Oita University [Approval No. 1414, May 20, 2019] was obtained. Written informed consent was obtained from the patient for the publication of this case report and associated images.

## 3. Discussion

The patient was taking two drugs before the fracture. Raloxifene hydrochloride is a selective estrogen receptor modulator and a bone resorption inhibitor. Raloxifene hydrochloride is mainly used in postmenopausal women. It is used for osteoporosis and reduces the incidence of vertebral fractures by 30%–50% [3]. However, no cumulative evidence has confirmed that it reduces the incidence of hip fractures or other nonvertebral fractures [3]. Raloxifene hydrochloride has been reported to be effective in the treatment of spinal osteoporosis, even in older women with renal failure [4]. Long-term administration of raloxifene hydrochloride reduces the risk of developing breast cancer but increases the incidence of venous thromboembolism [4]. On the contrary, bisphosphonate treatment which is a bone resorption inhibitor has been shown to prevent hip fractures in older people [5]. Despite evidence of the fracture-prevention effects of bisphosphonate [5, 6], only one study reported long-term treatment with alendronate, showing an increase in lumbar spine bone density after 10 years of treatment, and the lumbar spine bone density remained unchanged 5 years after its discontinuation [7]. On the contrary, there is an increased risk of atypical femoral fractures and osteonecrosis of the jaw [8]. The combination of raloxifene hydrochloride and bisphosphonate has been reported to be more effective for osteoporosis than either monotherapy and as safe as either alone [9, 10]. Although atypical femoral fractures are more common with bisphosphonate than with raloxifene hydrochloride [11], we cannot show the specific effects of both drugs on the fractures in this case because no study has reported the effects of atypical femoral fractures associated with the combination.

Stress fractures of the femoral neck caused by repetitive motion are rare, accounting for 7% of all fatigue fractures [12, 13]. Stress fractures of the femoral neck can be divided into two types: compression and transverse [14]. Compression-type fractures are more common among young people and caused by repeated compression stresses on the medial side (lower neck) of the femoral neck where physiological forces are applied. Transverse-type fractures occur on the lateral side (upper neck), where tension is applied, and fracture lines are evident on the radiograph at the time of the fracture.

Owing to an increasing number of the aged population worldwide, insufficiency fractures have recently gained attention. Several reports have described insufficiency fractures in the vertebral column of the spine, femoral head, and around the knee. Our patient was an older woman with severe osteoporosis, as evident from her bone mineral density and bone metabolism markers. Since there was no injury mechanism such as a fall, her fracture was classified as an insufficiency fracture.

Simultaneous bilateral stress fractures of the femoral neck are rare, except sports-related injuries. Insufficiency fractures of the bilateral femoral neck were reported in only five patients [15–19], mostly in young patients aged between 18 and 51 years not receiving medication for osteoporosis with no significant sex-specific difference (three women, two men). Osteochondrosis or osteoporosis was diagnosed in almost all cases after fractures. Our older patient had osteoporosis and was already receiving medication. This highlights a key difference between our case and previously reported cases.

Our case involved prolonged BP administration and prolonged fusion. Therefore, this case was considered an atypical femoral fracture. Atypical femoral fractures were initially defined as pathological fractures caused by excessive bone metabolism suppression due to long-term BP administration. While the involvement of specific drugs and underlying diseases has been discussed, the complete picture remains unclear [20]. BP administration has since been excluded from the definition, and atypical femoral fractures are now considered multifactorial, with possible involvement of race, body mass index, bone morphology, daily stress, drugs, and specific diseases [21]. Atypical fractures should be classified as fatigue fractures [22]. In our case, the histological findings of thickened bone trabeculae with multiple empty lacunae were similar to those of atypical fractures mentioned previously [22]. These findings indicate bone remodeling suppression.

The American Society of Bone Metabolism case definition of atypical femoral fractures [20] does not include femoral neck fractures. The major features described for atypical femoral fractures include the following: (1) located at any site along the femur from just distal to the lesser trochanter to just proximal to the supracondylar flare, (2) associated with no or minimal trauma (e.g., a fall from standing height or less), (3) transverse or short oblique configuration, (4) complete fractures extending through both cortices that may be associated with a medial spike with incomplete fractures involving only the lateral cortex, and (5) noncomminuted. In our patient, criteria (2), (4), and (5) were applicable and (3) if the fracture line perpendicular to the neck was considered a transverse fracture. Further, of the seven minor features—(1) localized periosteal reaction of the lateral cortex, (2) generalized increase in cortical thickness of the diaphysis, (3) prodromal symptoms such as dull or aching pain in the groin or thigh, (4) bilateral fractures and symptoms, (5) delayed healing, (6) comorbid conditions (e.g., vitamin D deficiency, rheumatoid arthritis, and hypophosphatasia), and (7) pharmaceutical agent use (e.g., BPs, glucocorticoids, proton pump inhibitors)—(1), (3), (4), (5), (6), and (7) were applicable in our case. Usually, the fracture line of an atypical fracture originates from the tensile side, not the compressive side. The fracture in our case occurred from the medial side of the femoral neck, defined as compression type on past reports of stress fractures [12, 13]. However, the stress distribution is completely different between the femoral neck and diaphysis essentially, and older patients such as our case have severe osteoporosis and different geometry of the femur compared with young ones [22]. Therefore, it is hard to believe that atypical fractures of the femoral neck always occur from the lateral side. Thus, our patient demonstrated all characteristics of an atypical fracture, except for the fracture site. Recently, periprosthetic and ulnar fractures with atypical femoral fracture characteristics have been reported [23, 24]. The possibility of this case being an atypical fracture could not be ruled out.

Case reports have inherent limitations, such as the inability to generalize, inability to establish a cause-effect relationship, danger of overinterpretation, and publication bias. In this case, we could not definitively confirm that the contemporaneous insufficiency fracture of the bilateral femoral neck was an atypical fracture. Even histological evaluation could not definitely diagnose an atypical fracture, stress fracture, or healing site for this case. However, imaging and histological findings, in this case, suggested atypical fractures, even though simultaneous insufficient fractures of the bilateral femoral neck can occur rarely.

To the best of our knowledge, this is the first case of simultaneous insufficiency fracture of the bilateral femoral neck in an older patient taking bisphosphonate. The patient was also vulnerable to atypical fractures based on clinical and histological findings that were consistent with the diagnostic criteria or findings of past reports. Our findings suggest that atypical fractures can occur in areas other than the femoral shaft.

## Data Availability

The datasets generated during and/or analyzed during the current study are available from the corresponding author on reasonable request.
